# Cumulative incidence of SARS-CoV-2 infection and associated risk factors among frontline health care workers in Paris: the SEROCOV cohort study

**DOI:** 10.1038/s41598-022-10945-y

**Published:** 2022-05-04

**Authors:** Pierre Hausfater, David Boutolleau, Karine Lacombe, Alexandra Beurton, Margaux Dumont, Jean-Michel Constantin, Jade Ghosn, Alain Combes, Nicolas Cury, Romain Guedj, Michel Djibré, Rudy Bompard, Sandie Mazerand, Valérie Pourcher, Linda Gimeno, Clémence Marois, Elisa Teyssou, Anne-Geneviève Marcelin, David Hajage, Florence Tubach

**Affiliations:** 1 Emergency Department, Hôpital Pitié-Salpêtrière, APHP, Sorbonne Université, 83 Boulevard de l’Hôpital, 75651 Paris Cedex 13, France; 2 GRC-14 BIOSFAST, UMR INSERM 1166, IHU ICAN, Sorbonne Université, Paris, France; 3 INSERM, Institut Pierre Louis d’Epidémiologie Et de Santé Publique (iPLESP), GH AP-HP, Hôpital Pitié-Salpêtrière, Service de Virologie, Sorbonne Université, Paris, France; 4 Infectious Disease Department, Sorbonne Université Hôpital Saint-Antoine, Paris, France; 5 Service de Pneumologie-Médecine Intensive Réanimation, Hôpital Pitié-Salpêtrière, APHP, Sorbonne Université Inserm UMRS Neurophysiologie Respiratoire Expérimentale et Clinique, Sorbonne Université, Paris, France; 6 Department of Anaesthesiology and Critical Care, GRC 29, AP-HP, DMU DREAM, Hôpital Pitié-Salpêtrière, GH APHP, Sorbonne Université, Paris, France; 7 AP-HP, Nord, Service des Maladies Infectieuses et Tropicales, Hôpital Bichat-Claude Bernard, and Université de Paris, INSERM, UMR 1137 IAME, Paris, France; 8 INSERM, UMRS_1166-ICAN, Institute of Cardiometabolism and Nutrition, and Service de Médecine Intensive-Réanimation, Institut de Cardiologie, GH APHP Sorbonne Université Hôpital Pitié–Salpêtrière, Sorbonne Université, Paris, France; 9 Emergency Department, APHP, Sorbonne Université Hôpital Saint-Antoine, Paris, France; 10 Pediatric Emergency Deparment, APHP Hôpital Armand Trousseau-Sorbonne Université, Faculté de Médecine, Paris, France; 11 Centre of Research in Epidemiology and Statistics-CRESS, INSERM, Université de Paris, 75004 Paris, France; 12 Service de Médecine Intensive Réanimation, APHP, Sorbonne Université Hôpital Tenon, Paris, France; 13 Emergency Department, Hôpital Tenon, APHP, Sorbonne Université, Paris, France; 14 Service de Médecine Intensive-Réanimation, APHP, Sorbonne Université Hôpital Saint-Antoine, Paris, France; 15 AP-HP, Hôpitaux Universitaires Pitié-Salpêtrière Charles Foix, Service de Maladies Infectieuses et Tropicales, INSERM 1136, Institut Pierre Louis d’Epidémiologie et de Santé Publique, Sorbonne Université, 75013 Paris, France; 16 APHP, Unité de Recherche Clinique Pitié Salpêtrière Charles Foix, Sorbonne Université, 75013 Paris, France; 17 Unité de Médecine Intensive Réanimation Neurologique, Département de Neurologie, DMU Neurosciences, APHP, Sorbonne Université Hôpital Pitié-Salpêtrière, Paris, France; 18 Département de Santé Publique, INSERM, Institut Pierre Louis d’Epidémiologie et de Santé Publique, AP-HP, Hôpital Pitié Salpêtrière, Sorbonne Université, CIC-1422, 75013 Paris, France

**Keywords:** Virology, Microbiology, Health occupations, Risk factors, Infectious diseases

## Abstract

With the COVID-19 pandemic, documenting whether health care workers (HCWs) are at increased risk of SARS-CoV-2 contamination and identifying risk factors is of major concern. In this multicenter prospective cohort study, HCWs from frontline departments were included in March and April 2020 and followed for 3 months. SARS-CoV-2 serology was performed at month 0 (M0), M1, and M3 and RT-PCR in case of symptoms. The primary outcome was laboratory-confirmed SARS-CoV-2 infection at M3. Risk factors of laboratory-confirmed SARS-CoV-2 infection at M3 were identified by multivariate logistic regression. Among 1062 HCWs (median [interquartile range] age, 33 [28–42] years; 758 [71.4%] women; 321 [30.2%] physicians), the cumulative incidence of SARS-CoV-2 infection at M3 was 14.6% (95% confidence interval [CI] [12.5; 16.9]). Risk factors were the working department specialty, with increased risk for intensive care units (odds ratio 1.80, 95% CI [0.38; 8.58]), emergency departments (3.91 [0.83; 18.43]) and infectious diseases departments (4.22 [0.92; 18.28]); current smoking was associated with reduced risk (0.36 [0.21; 0.63]). Age, sex, professional category, number of years of experience in the job or department, and public transportation use were not significantly associated with laboratory-confirmed SARS-CoV-2 infection at M3. The rate of SARS-CoV-2 infection in frontline HCWs was 14.6% at the end of the first COVID-19 wave in Paris and occurred mainly early. The study argues for an origin of professional in addition to private life contamination and therefore including HCWs in the first-line vaccination target population. It also highlights that smokers were at lower risk.

**Trial registration** The study has been registered on ClinicalTrials.gov: NCT04304690 first registered on 11/03/2020.

## Introduction

The dynamics of the COVID-19 pandemic has deeply affected health services in organizations and potentially exposed healthcare workers (HCWs) to increased risk of SARS-CoV-2 infection. Since the emergence of this new human coronavirus in December 2019^1^, knowledge of the modes of transmission has improved weekly, leading to the adaptation of HCWs’ personal protective equipment (PPE). Hence, the risk of SARS-CoV-2 infection among HCWs has been of major concern for frontline institutions, to prevent COVID-19-associated work disruption (at a time when care resources were critical) and HCW-to-patient contamination. In France, SARS-CoV-2-positive RT-PCR tests were first reported in imported cases on January 24, 2020; the generalized lockdown began on March 17 and emergency room visits for possible COVID-19 peaked on week 13, decreasing thereafter, which led the French government to ease lockdown restrictions on May 11.

Although some cross-sectional studies have reported SARS-CoV-2 seroprevalence among HCWs being close to that of the general population^2–7^, little is known about the dynamics of SARS-CoV-2 contamination related to the epidemic waves and the risk factors in this population.

In the ascendant phase of the first COVID-19 wave in France, we initiated a multicenter prospective cohort study to estimate the cumulative incidence of laboratory-confirmed SARS-CoV-2 infection over the early phase of this outbreak among frontline public hospital HCWs in Paris and identify the risk factors (SEROCOV, ClinicalTrials.gov NCT04304690, first registered on 11/03/2020).

## Patients and methods

### Setting, design and participants

The SEROCOV multicenter prospective cohort study was conducted in 4 adult hospitals (Bichat, Pitié-Salpêtrière, Saint-Antoine, Tenon) and 1 pediatric hospital (Trousseau) of the Assistance Publique-Hôpitaux de Paris (AP-HP) network. Participants were included from March 16 to April 24 2020, with a 3-month follow-up. Bichat and Pitié-Salpêtrière hospitals are dedicated referent hospitals for emerging biological risk at AP-HP and primarily received all COVID-19 confirmed cases, from the epidemic onset in Paris until mid-March 2020, when all AP-HP hospitals were positioned to take care of COVID-19 patients.

The SEROCOV study was approved by the ethics committee (CPP Sud-Ouest et Outre-Mer I, approval no. 2-20-023 id7257) and all participants signed informed consent before inclusion. We also confirm that all experiments were performed in accordance with relevant guidelines and regulations.

All HCW staff (doctors—either senior physicians or residents—nurses, care assistants, laboratory technicians and other personals in contact with patients) who worked in COVID-19 frontline departments (emergency, infectious diseases, intensive care units and virology laboratory) of the selected hospitals were informed about the study and asked to participate. Staff who were not active during the inclusion period were not eligible. After signing informed consent, each participant underwent venous blood sampling for SARS-CoV-2 serology and was asked to complete a self-reported paper-based questionnaire on baseline characteristics and pre-inclusion symptoms suggestive of COVID-19. Thereafter and up to 3 months after inclusion, the HCW participants received a weekly reminder to repeat and fill the survey on symptoms and, in case of symptoms, asked for a nasopharyngeal swab for SARS-CoV-2 RT-PCR. The survey was collected on a paper form which was stored in each department and made available all along the follow-up period for the participants. At month 1 (M1) and M3 after inclusion, venous blood sampling was again systematically proposed for serology testing.

### Anti-SARS-CoV-2 IgG assay for serology testing

Blood samples were sent immediately to the virology department of each participating hospital and centrifuged, and EDTA plasma was frozen at − 20 °C until tested by batch in the Pitié-Salpêtrière virology laboratory. Plasma samples were analyzed on the Abbott Architect platform with the Abbott SARS-CoV-2 IgG assay, targeting the viral nucleoprotein^8^. Results were interpreted as recommended by the manufacturer: an index value < 0.5 was considered negative, 0.5–1.4 weak positive, and ≥ 1.4 positive. When SARS-CoV-2 infection was suspected in an HCW during the study period, the COVID-19 diagnosis was established by RT-PCR on a nasopharyngeal swab with the Cobas SARS-CoV-2 kit (Roche Diagnostics) or RealStar SARS-CoV-2 RT-PCR kit (Altona), as previously reported^9^.

### Outcomes

The primary outcome was laboratory-confirmed SARS-CoV-2 infection at M3 (i.e., positive serology results [positive or weak positive result on SARS-CoV-2 IgG assay] and/or a positive RT-PCR result on an unplanned nasopharyngeal swab at any time during the survey). Secondary outcomes were positive serology for SARS-CoV-2 (considering weak positive serology as positive) at M0, M1 and M3.

### Covariates

Baseline characteristics collected were demographics, smoking status (current, former or non-smoker), public transportation use, and job-related data (hospital, type of ward [emergency, infectious diseases, intensive care unit and virology laboratory]), professional category, number of years of experience in the job or department, and night or day shift. Physicians (senior or medical students, including residents), nurses and care assistants who had direct close contacts with COVID-19 patients were considered high-risk HCWs (in contrast to nurses managers for example).

At baseline and up to 3 months after inclusion, participants completed a self-reported questionnaire weekly on symptoms suggestive of COVID-19 and as appropriate, SARS-CoV-2 RT-PCR results, the diagnosis retained, and COVID-related sick days and/or hospitalization.

At M3, adherence to PPE recommendations during the study period was reported on a 5-point Likert scale (from “never” to “systematically”).

### Statistical analysis

The number of eligible HCWs in participating services was estimated at approximately 1,000 at the time this study was designed. This planned sample size allowed for estimating a cumulative incidence of laboratory-confirmed SARS-CoV-2 infection at M3 of 5% (95% confidence interval [CI] [3.7; 6.5]), a cumulative incidence of 10% (8.2; 12), and a cumulative incidence of 20% (17.6; 22.6).

Staff characteristics are reported as percentages for categorical variables and median (interquartile range [IQR]) for continuous variables.

Multiple imputation was used to replace missing serology testing at M0, M1 or M3. Briefly, 10 copies of the dataset were created with the missing values replaced by imputed values based on observed data including observed serology results and characteristics of participants (including age, sex, job-related data, smoking status, public transportation use, adherence to PPE recommendations and occurrence of ageusia and/or anosmia over the study period). Each dataset was then analyzed by using standard statistical methods, and the results from each dataset using the statistical methods described below were pooled into a final result by using Rubin’s rule^10^.

For binomial proportions, confidence intervals were estimated by the Wilson method for multiple imputation^11^.

Risk factors of laboratory-confirmed SARS-CoV-2 infection at M3 were assessed in the whole cohort by univariate and multivariate logistic regression. Baseline variables included in the multivariate model were defined a priori (age, sex, job-related data, public transportation use and smoking status), and no variable selection was performed. A second logistic model was developed for the same outcome among high-risk HCWs only (i.e., excluding virology staff and jobs rated as “other”) and accounting for adherence to PPE recommendations. Finally, risk factors of laboratory-confirmed SARS-CoV-2 infection at inclusion (M0) were evaluated by the same approach.

All the analyses were computed at a two-sided α level of 5% with R software, version 4.0.0.

### Ethics approval

The SEROCOV study was approved by the ethics committee (CPP Sud-Ouest et Outre-Mer I, approval no. 2-20-023 id7257).

## Results

From March 16 to April 24, 2020, 1,177 HCWs were included; 115 were excluded because they did not work in the participating departments, so 1062 HCWs remained for analysis. The participation rate was 77% for medical staff and 59.5% for nurses/care assistants. The main characteristics of the study population are in Table 1. Median [IQR] age was 33 [28–42] years, 758 (71.4%) were women, 741 (69.8%) were nurses/care assistants staff (including 405 [38.1%] nurses) and 321 (30.2%) were physicians (including students); 380 (35.8%) were working in an emergency department, 282 (26.6%) in an infectious diseases unit, 355 (33.4%) in an intensive care unit and 45 (4.2%) in a virology laboratory. Among the participants, 250 (26.9%) reported currently smoking, and 387 (36.4%) used public transportation.

**Table 1:** Baseline characteristics of the study population according to SARS-CoV-2 serology at inclusion.

	Negative SARS-CoV-2 serology (N = 999)	Positive SARS-CoV-2 serology (N = 61)	Total^a^ (N = 1062)
Age	33 [28–42.5]	31 [26–38]	33 [28–42]
Women	717 (71.8%)	40 (65.6%)	758 (71.4%)
**Professional category**			
Senior physicians	208 (20.8%)	12 (19.7%)	221 (20.8%)
Medical students (including residents)	89 (8.9%)	11 (18%)	100 (9.4%)
Care assistants	197 (19.7%)	6 (9.8%)	203 (19.1%)
Nurses	378 (37.8%)	27 (44.7%)	405 (38.1%)
Others^b^	127 (12.7%)	5 (8.2%)	133 (12.5%)
**Working department**			
Emergency department	372 (37.2%)	7 (11.5%)	380 (35.8%)
Infectious diseases unit	239 (23.9%)	43 (70.5%)	282 (26.6%)
Intensive care unit	343 (34.3%)	11 (18%)	355 (33.4%)
Virology laboratory	45 (4.5%)	0 (0%)	45 (4.2%)
Working in a COVID-free unit	80 (8%)	2 (3.3%)	82 (7.7%)
Working in a referent hospital for emerging biological risk	519 (52%)	32 (52.5%)	552 (52%)
Night shift	123 (12.3%)	5 (8.2%)	129 (12.2%)
Missing data, No	1	0	1
Experience in the job (months)	84 [36–180]	72 [30–168]	84 [36–180]
Missing data, No	5	0	5
Experience in the department (months)	36 [10–84]	24 [6–72]	36 [9–84]
Missing data, No	14	0	14
Public transportation use	369 (40.5%)	18 (40.9%)	387 (40.5%)
Missing data, No	88	17	105
Active smoker	246 (27.8%)	4 (8.9%)	250 (26.9%)
Missing data, No	115	16	132
Symptoms suggestive of COVID-19 before inclusion	125 (13.3%)	29 (50%)	154 (15.4%)
Missing data, No	57	3	
If yes: fever	29 (23.2%)	13 (44.8%)	42 (27.3%)
Anosmia and/or agueusia	10 (8%)	14 (48.3%)	24 (15.6%)
Respiratory symptoms	56 (44.8%)	13 (44.8%)	69 (44.8%)
Gastrointestinal symptoms	25 (20%)	2 (6.9%)	27 (17.5%)
**Adherence to PPE recommendations over the study period**^**c**^			
Wear a surgical mask	829 (93%)	40 (85%)	870 (93.1%)
Wear an FFP2 mask to take nasopharyngeal swabs	521 (78%)	28 (82%)	549 (77.8%)
Wear an N95 mask to handle a confirmed COVID-19 case	578 (74%)	24 (56%)	603 (73.2%)
If wearing a mask (surgical or FFP2), change every 4 h	570 (65%)	22 (47%)	592 (63.9%)
Hydro-alcoholic friction or hand washing before and after each patient contact	831 (99%)	45 (98%)	877 (98.5%)

Data are n (%) for qualitative variables and median [interquartile range] for quantitative variables.

^a^HCWs with positive, negative or missing (n = 2) serology at inclusion, thus 2 more individuals than the sum of the 2 other columns.

^b^Others include (but not limited to): secretaries, nurse’s managers, social workers.

^c^Collected at the end of the study on a 5-point Likert scale (0, never, to 5, systematically), reported as the proportion of HCWs rating 4 or 5; PPE: personal protective equipment.

**Figure 1:**
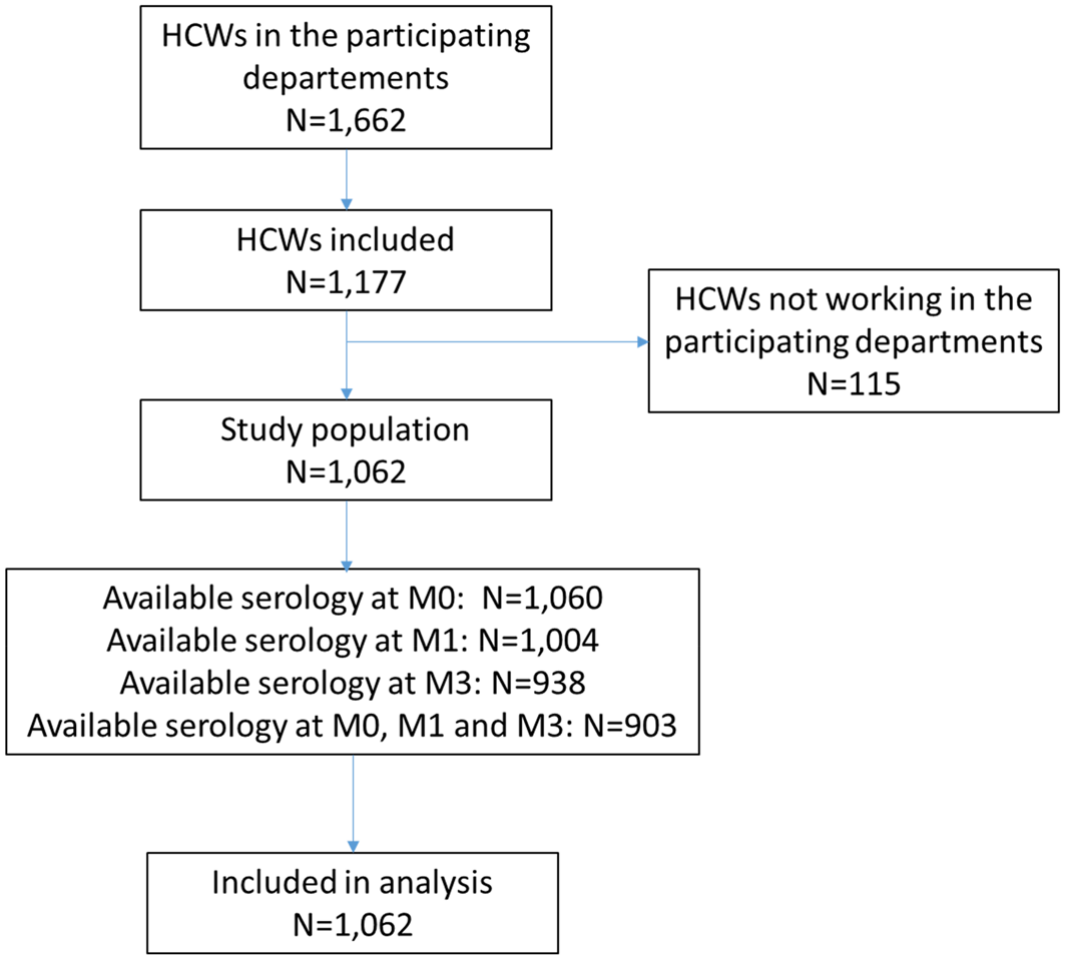
Flow chart. M, month.

**Figure 2:**
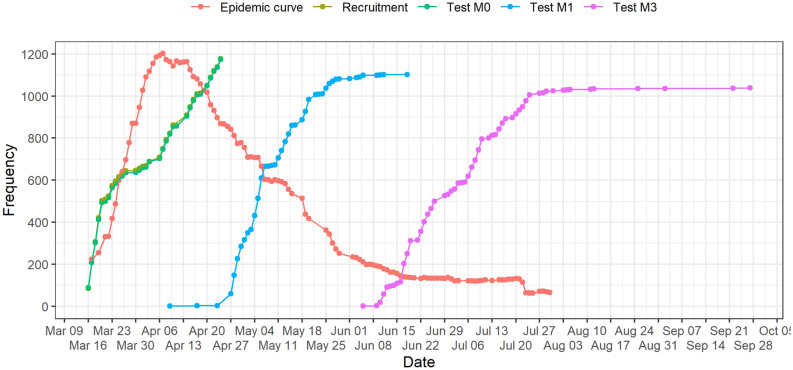
Dates of inclusion (recruitment) and serology tests according to the epidemic curve (in patients with documented COVID-19 in AP-HP hospital network).

Overall, 1060 (99.8%) participants underwent baseline serology testing at inclusion, 1004 (94.5%) at M1, and 938 (88.3%) at M3; 903 (85%) had a SARS-CoV-2 serology result available for the 3 samples (Fig. 1). At M3, during the whole study period, 93.0% of HCWs reported that they wore a surgical mask, 77.8% wore an N95 mask when performing high-risk tasks (nasopharyngeal swab), and 98.5% washed hands and/or used hydro-alcoholic hand friction before and after each contact with a patient (Table 1). The dates of inclusion and follow-up serologies according to the epidemic curve are in Fig. 2.

At baseline, 154/1062 (15.4%) HCWs with available data reported having symptoms suggestive of COVID-19 before inclusion, mainly fever (n = 42), myalgia (n = 34), headache (n = 54), ageusia and/or anosmia (n = 24), cough (n = 55), respiratory signs (n = 69), and gastrointestinal signs (n = 27) (Table 1).

At inclusion, 61 (5.8%) HCWs had positive SARS-CoV-2 serology results: 23 (7.2%) physicians and 38 (5.1%) nurses/care assistants staff. Reports of previous symptoms suggestive of COVID-19 were more frequent for HCWs with positive than negative serology at inclusion (50% vs 13.3%; p < 0.0001), particularly symptoms very suggestive of COVID-19 such as ageusia/anosmia (48.3% vs 8%). Conversely, 50% of participants with positive serology at inclusion reported no previous symptoms.

Because of symptoms suggestive of COVID-19, 33 participants submitted an intermediate nasopharyngeal swab for SARS-CoV-2 RT-PCR between inclusion and M3: 14 (42.4%) had a positive result, 2 with negative results at all serology points. Among the 134 participants with seropositive results at inclusion or M1, 11 (8.2%) had negative serology results at M3, including 8 with positive results at inclusion.

### Outcomes

**Figure 3:**
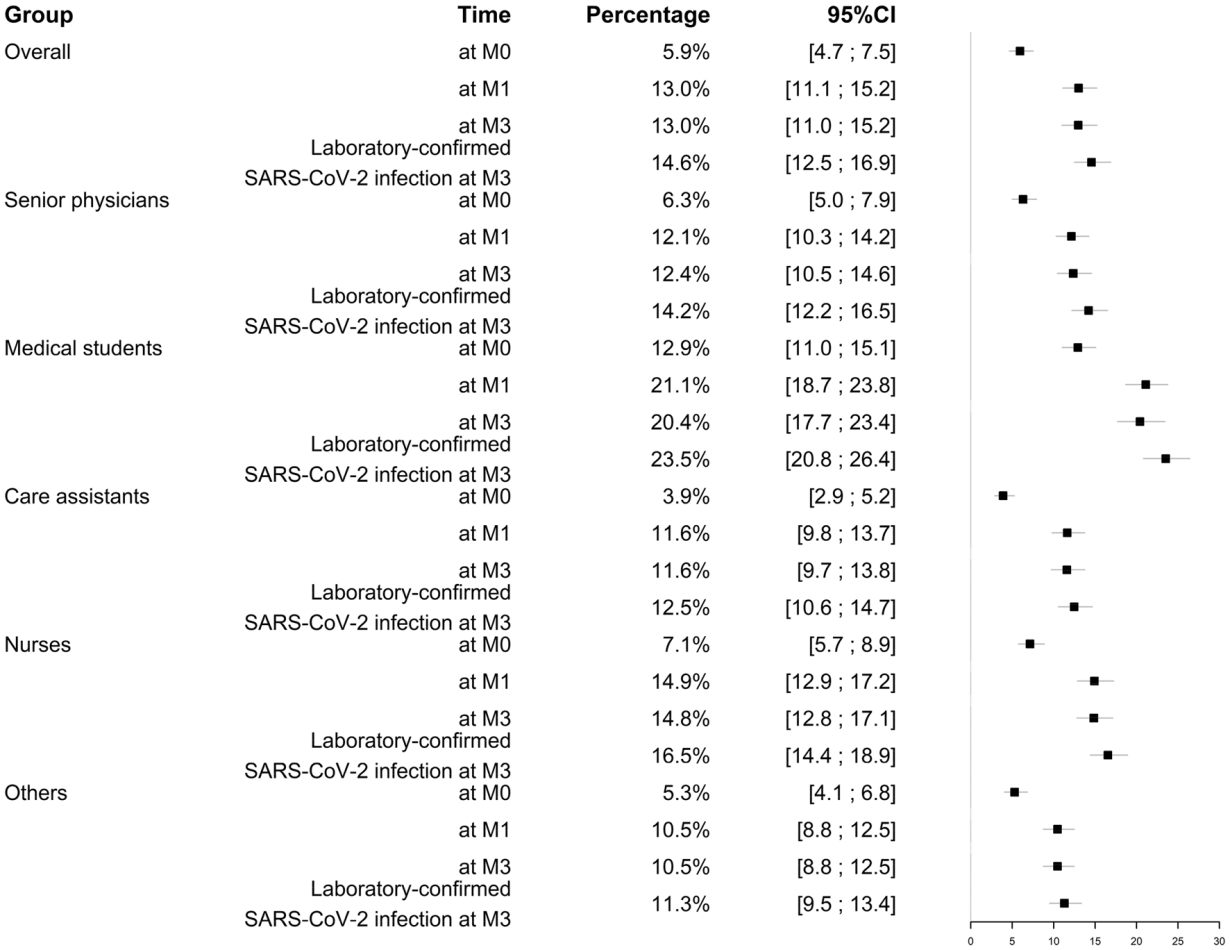
Laboratory-confirmed infection with SARS-CoV-2 at month 3 (M3) and seroprevalence at M0, M1 and M3 according to professional category.

The estimated cumulative incidence of laboratory-confirmed SARS-CoV-2 infection at M3 after imputation of missing data was 14.6% (95% CI [12.5; 16.9]), and the difference across professional categories was not statistically significant, although the incidence was highest for medical students (23.5% [20.8; 26.4]) and higher in nurses than in care assistants (Table 2 and Fig. 3).

**Table 2:** Laboratory-confirmed infection with SARS-CoV-2 at month 3 (M3) and seroprevalence at M0, M1 and M3 after imputation of missing data, according to professional category.

	All	Senior physicians	Medical students	Care assistants	Nurses	Others	p value*
**Serology positive**							
At M0	5.9% [4.7; 7.5]	6.3% [5.0; 7.9]	12.9% [11.0; 15.1]	3.9% [2.9; 5.2]	7.1% [5.7; 8.9]	5.3% [4.1; 6.8]	0.06
At M1	13.0% [11.1; 15.2]	12.1% [10.3; 14.2]	21.1% [18.7; 23.8]	11.6% [9.8; 13.8]	14.9% [12.9; 17.2]	10.5% [8.8; 12.5]	0.13
At M3	13.0% [11.1; 15.2]	12.4% [10.5; 14.6]	20.4% [17.7; 23.4]	11.6% [9.7%; 13.8]	14.8% [12.8; 17.1]	10.5% [8.8; 12.5%]	0.21
Laboratory-confirmed SARS-CoV-2 infection at M3	14.6% [12.5; 16.9]	14.2% [12.2; 16.5]	23.5% [20.8; 26.4]	12.5% [10.6; 14.7]	16.6% [14.4; 18.9]	11.3% [9.5; 13.4]	0.12

Data are % (95% confidence interval). Others include (but not limited to): secretaries, nurse’s managers, social workers.

*Comparison across professional categories.

The estimated seroprevalence after imputation of missing data at M0, M1, and M3 was 5.9% [4.7; 7.5], 12.9% [10.9; 15.1] and 13.0% [11.1; 15.2], with the same trend across professional categories but not statistically significant.

Of the 147 participants with laboratory-confirmed SARS-CoV-2 infection at M3, only 88 (59.9%, 95% CI [51.47; 67.85]) reported symptoms suggestive of COVID-19 before or during the study period. Among the 416 participants with negative serology at M0 and reporting symptoms suggestive of COVID-19 during the study period, in those with and without documented SARS-CoV-2 infection, 58.7% vs 13.3% reported sick leave related to these symptoms (p < 0.0001). Among the 147 participants with laboratory-confirmed infection by SARS-CoV-2 at M3, 3 were hospitalized for COVID-19 and none died.

### Risk factors for SARS-CoV-2 positive serology at inclusion

After multiple imputation for missing data, on multivariate analysis (Table 3), risk factors for SARS-CoV-2 positive serology at inclusion (i.e., during the increasing phase of first epidemic wave) were the department where the participant worked, with increased risk for intensive care units (OR 1.29, 95% CI [0.47; 3.51]) and particularly infectious disease units (6.61 [2.64; 16.54]) versus emergency departments (virology laboratory was not included because no virology staff were positive at inclusion). Being a current smoker reduced the risk (0.28 [0.10; 0.82]). Age, sex, using public transportation, professional category, working in a referent hospital for emerging biological risk, number of years of experience in the job or department, and night shift were not significantly associated with baseline positive serology.

**Table 3:** Risk factors of laboratory-confirmed SARS-CoV-2 infection at inclusion (after imputation of missing data; N = 1017).

	Univariate analysis			Multivariate analysis		
	OR	[95% CI]	p value	OR	[95% CI]	p value
Age	0.99	[0.96; 1.02]	0.42	0.98	[0.95; 1.01]	0.26
Women	0.76	[0. 44; 1. 32]	0.33	0.60	[0.30; 1.23]	0.16
Working in a referent hospital for emerging biological risk	1.11	[0.66; 1.87]	0.69	1.08	[0.56; 2.09]	0.83
**Working department**			< 0.0001			< 0.0001
Emergency department	1			1		
Intensive care unit	1.68	[0.64; 4.39]		1.29	[0.47; 3.51]	
Infectious diseases department	9.46	[4.18; 21.40]		6.61	[2.64; 16.54]	
**Professional category**			0.08			0.50
Physicians	1			1		
Medical students	2.17	[0.92; 5.11]		1.62	[0.42; 6.28]	
Care assistants	0.51	[0.19; 1.40]		1.18	[0.38; 3.63]	
Nurses	1.20	[0.59; 2.41]		1.86	[0.77; 4.50]	
Others	0.89	[0.30; 2.60]		0.78	[0.22; 2.69]	
Experience in the department ≥ 2 months	0.71	[0.41; 1.23]	0.22	1.27	[0.49; 3.25]	0.62
Experience in the job ≥ 12 months	1.20	[0.47; 3.07]	0.71	0.94	[0.25; 3.56]	0.92
Night shift	0.61	[0.24; 1.56]	0.30	0.52	[0.12; 2.31]	0.39
Public transportation use	1.11	[0.62; 2.02]	0.65	0.99	[0.51; 1.94]	0.84
Active smoker	0.25	[0.09; 0.70]	0.009	0.28	[0.10; 0.82]	0.02

HCWs from virology department were excluded because none had a positive serology result at inclusion.

Others include (but not limited to): secretaries, nurse’s managers, social workers.

*OR* odds ratio, *CI* confidence interval.

### Risk factors for laboratory-confirmed SARS-CoV-2 infection at M3

After multiple imputation for missing data, on multivariate analysis (Table 4), risk factors for laboratory-confirmed SARS-CoV-2 infection at M3 (i.e., at the end of the first epidemic wave) were the department where the participant worked, with increased risk for intensive care units (OR 1.80, 95% CI [0.38; 8.58]) and particularly emergency departments (3.91 [0.83; 18.43]) and infectious diseases units (4.22 [0.92; 18.28]) versus the virology laboratory. Being a current smoker reduced the risk (0.36 [0.21; 0.3]). Age, sex, use of public transportation, professional category, working in a referent hospital for emerging biological risk, number of years of experience in the job or department, or night shift were not significantly associated with laboratory-confirmed SARS-CoV-2 infection at M3.

**Table 4:** Risk factors of laboratory-confirmed SARS-CoV-2 infection at M3 (after imputation of missing data; N = 1,062)).

	Bivariate analysis			Multivariable analysis		
	OR	[95% CI]	p value	OR	[95% CI]	p value
Age	0.98	[0.97; 1.00]	0.06	0.99	[0.97; 1.01]	0.41
Women	1.05	[0.70; 1.56]	0.79	1.04	[0.65; 1.67]	0.85
Working in a referent hospital for emerging biological risk	0.90	[0.63; 1.28]	0.57	1.16	[0.76; 1.77]	0.49
**Working department**			0.0002			0.003
Virology laboratory	1			1		
Intensive care unit	2.07	[0.47; 9.02]		1.80	[0.38; 8.58]	
Emergency department	4.10	[0.97; 17.39]		3.91	[0.83; 18.43]	
Infectious diseases department	5.65	[1.33; 23.09]		4.22	[0.92; 18.28]	
**Professional category**			0.12			0.38
Senior physicians	1			1		
Medical students	1.79	[0.93; 3.44]		1.69	[0.68; 4.18]	
Care assistants	0.85	[0.47; 1.53]		0.99	[0.50; 1.96]	
Nurses	1.24	[0.77; 2.00]		1.49	[0.85; 2.63]	
Others^a^	0.71	[0.35; 1.42]		0.87	[0.40; 1.87]	
Experience in the department ≥ 12 months	0.78	[0.53; 1.16]	0.23	1.14	[0.60; 2.17]	0.70
Experience in the job ≥ 12 months	0.86	[0.48; 1.55]	0.63	0.97	[0.42; 2.24]	0.83
Night shift	1.03	[0.60; 1.75]	0.88	1.00	[0.52; 1.90]	0.91
Public transportation use	1.21	[0.84; 1.75]	0.32	1.17	[0.79; 1.75]	0.44
Active smoker	0.38	[0.22; 0.66]	0.0005	0.36	[0.21; 0.63]	0.0003

*OR* odds ratio, *CI* confidence interval.

^a^Others include (but not limited to): secretaries, nurse’s managers, social workers.

In the subgroup of high-risk HCWs (i.e., physicians, nurses and care assistants in clinical wards), adjustment on adherence to PPE recommendations did not change these results (Table 5).

**Table 5:** Risk factors of laboratory-confirmed SARS-CoV-2 infection at month 3 in high-risk healthcare workers, accounting for adherence to personal protective equipment recommendations (after imputation of missing data; N = 916)).

	Bivariate analysis			Multivariable analysis		
	OR	[95% CI]	p value	OR	[95% CI]	p value
Age	0.99	[0.97; 1.01]	0.24	0.99	[0.97; 1.016]	0.51
Women	1.07	[0.70; 1.62]	0.74	0.99	[0.60; 1.65]	0.89
Working in a referent hospital for emerging biological risk	0.97	[0.67; 1.42]	0.80	1.20	[0.77; 1.86]	0.43
**Working department**			0.0001			0.01
Intensive care unit	1			1		
Emergency department	1.93	[1.17; 3.18]		1.86	[1.05; 3.31]	
Infectious diseases department	3.10	[1.85; 5.22]		2.38	[1.30; 4.35]	
**Professional category**			0.17			0.25
Physicians	1			1		
Medical students	1.80	[0.93; 3.49]		1.76	[0.69; 4.51]	
Care assistants	0.82	[0.45; 1.47]		1.21	[0.58; 2.50]	
Nurses	1.19	[0.74; 1.92]		1.75	[0.96; 3.18]	
Experience in the department ≥ 12 months	0.77	[0.51; 1.16]	0.22	1.26	[0.63; 2.52]	0.55
Experience in the job ≥ 12 months	0.87	[0.48; 1.56]	0.65	0.92	[0.39; 2.18]	0.77
Night shift	0.93	[0.54; 1.61]	0.80	0.93	[0.47; 1.83]	0.83
Public transportation use	1.34	[0.91; 1.99]	0.14	1.28	[0.83; 1.95]	0.27
Active smoker	0.37	[0.21; 0.66]	0.0006	0.36	[0.20; 0.64]	0.0006
**Adherence to PPE recommendations over the study period**^**a**^						
Wear a surgical mask < 4	0.70	[0.29; 1.67]	0.44	0.57	[0.21; 1.57]	0.28
Wear an N95 mask to take nasopharyngeal swabs < 4	1.02	[0.61; 1.71]	0.71	1.02	[0.56; 1.87]	0.71
Wearing an N95 mask to handle a confirmed COVID-19 case < 4	1.58	[0.99; 2.51]	0.06	1.36	[0.78; 2.35]	0.29
If wearing a mask (surgical or FFP2), change every 4 h < 4	1.35	[0.90; 2.04]	0.16	1.32	[0.82; 2.13]	0.26

Data are n (%) for qualitative variables; median [interquartile range] for quantitative variables; OR: odds ratio; CI: confidence interval.

^a^Collected at the end of the study on a 5-point Likert scale (0 = never to 5 = systematically), reported as the proportion of HCW rating 4 or 5; PPE: personal protective equipment.

## Discussion

We conducted this multicenter prospective cohort study among frontline HCWs to investigate SARS-CoV-2 infection in HCWs during the first COVID-19 epidemic wave in Paris, France. We report a 5.8% baseline (early phase) SARS-CoV-2 seroprevalence rate together with a 3-month 14.6% rate of laboratory-confirmed SARS-CoV-2 infection (end of first wave). Most infections occurred very early, that is, diagnosed at M1. Among the identified risk factors was the working department, infectious disease units being at highest risk at the very early phase, then emergency departments during the epidemic peak, with the same level of risk for both at the end of the wave and intensive care units with an intermediate risk, with the virology laboratory staff as reference. Current smokers were at reduced risk. Demographics, other job-related characteristics, adherence to PPE, and use of public transportation were not significantly associated with SARS-CoV-2 infection. To our knowledge, our study is the largest reporting the longitudinal evolution of anti-SARS-CoV-2 antibodies in HCWs over a 3-month period.

The seroprevalence in the general population estimated in the SAPRIS study^12^ in the same geographical area (Greater Paris) in May 2020, corresponding to M1 in the SEROCOV study, was significantly lower than in the SEROCOV first-line HCWs: 6.4% in the 20- to 59-year-old population of the SAPRIS study (using a similar anti-nucleoprotein assay and considering weak positive as positive as in SEROCOV) versus 13.3% in SEROCOV (*p* < 0.0001). Previous published HCW surveys reported seroprevalence ranging from 3.4 to 38%^13,14^. However, the heterogeneity in type of HCW tested, type of assay used, period of testing and countries with incomparable SARS-CoV-2 epidemic burden precludes a face-to-face comparison. In a multicenter cross-sectional study in the New York city area, Moscola et al. reported a 13.7% prevalence (95% CI [13.4; 14.0]) of SARS-CoV-2 antibodies in 40,329 HCWs, a rate similar to that among adults randomly tested in New York state (14.0%)^3^. However, in another cross-sectional study in Roslyn, New York, in one hospital, employees had a significantly lower serology-positive rate than the general population (9.9% vs 16.7%, *P* < 0.001)^4^. Stubblefield et al. reported a 7.6% seroprevalence among 249 frontline HCWs during the first month of epidemic in Nashville, Tennessee, whereas Jespersen et al. reported a 3.4% seroprevalence (95% CI [2.5; 3.8]) among 17,971 HCWs, administrative personnel, pre-hospital services and specialist practitioner clinics in Denmark, with high geographical seroprevalence variation (from 1.2 to 11.9%)^6,7^. In the United States, in a Multistate Hospital Network, Self et al. reported 6.0% positive serology in front-line HCWs^5^. Among the 194 participants with positive results, 56 (29%) reported no symptoms in the previous weeks. In our study, this asymptomatic proportion was 50% among participants with positive serology at inclusion. Conversely, we confirm that anosmia and/or ageusia during the epidemic wave was a discriminative clinical sign, although with low sensitivity. This result confirms the Belgian monocentric study findings reporting of anosmia associated with an OR for positive serology of 7.78 ([95% CI [5.22; 11.53])^2^.

Most HCW contaminations occurred very early during the epidemic wave, almost half documented at baseline (positive serology: 5.9%, 95% CI [4.7; 7.5]) and 12.9% at M1 (corresponds to infections during the ascendant phase of the epidemic wave as shown in Fig. 2). The kinetics of these infections probably reflects both infections in the private sphere, following the epidemic curve of the general population, but also the effectiveness of better adherence to protective measures in professional activity related to an enforcement of PPE and standard measures as the epidemic progressed. The family and friends or professional origin of HCW contamination is still debated^15^. In a single center study in Belgium of 3056 HCWs (tested with an IgG/IgM rapid lateral flow assay), the OR for positive serology was 3.15 (95% CI [2.33; 4.25]) when reporting household contact with a suspected or confirmed COVID-19 case^2^. Conversely, a large study in Denmark of 17,971 hospital staff (based on the adjusted seroprevalence according to living and working places) found risk of SARS-CoV-2 positive serology associated with workplace rather than place of living^7^.

Recently, a large survey of HCWs in the United Kingdom reported anti-spike or anti-nucleocapsid IgG antibodies associated with substantially reduced risk of SARS-CoV-2 reinfection and highlighted that individual HCWs middle-/long-term protection by post-COVID self-immunization is a major concern^16^. Our data support the persistence of SARS-CoV-2 antibodies at 3 months: only 11 (8%) participants with positive serology had later negative results. Controversial results were reported by Patel et al. for 249 HCWs with a positive serology rate decreasing from 7.6% at baseline to 3.2% at 60 days, whereas Gudbjartsson et al. showed no decline in anti-SARS-CoV-2 antibodies at 4 months after COVID-19 diagnosis^17,18^.

Workers in first-line clinical departments (infectious diseases, emergency, intensive care unit) were at higher risk of laboratory-confirmed SARS-CoV-2 infection as compared with virology laboratory staff (Table 3). In a large study in Denmark, Jespersen et al. reported the highest adjusted seroprevalence in emergency departments: 29.7% (95% CI [23.1; 37.6]); departments with no or limited patient contact had the lowest seroprevalence: 1.79% (0.31; 3.90)^7^. From January to mid-March 2020, infectious disease departments and intensive care units directly admitted patients with suspected COVID-19 from home or the general practitioner’s office, bypassing the emergency department, and were therefore initially more exposed to SARS-CoV-2 than emergency department workers. This situation may explain the association of SARS-CoV-2 positive serology at inclusion with working in intensive care units (OR 1.2 [95% CI (0.47; 3.51)]) and especially infectious diseases units (6.61, [2.64; 16.54]) versus emergency departments (Table 3). Virology laboratory staff, although handling numerous SARS-CoV-2-contaminated samples, are probably more concerned and stricter about PPE enforcement to prevent contamination and had no staff with positive serology at inclusion, together with the lowest seroprevalence rate at 3 months. Similarly, as compared with emergency department staff, intensive care unit staff were at intermediate risk, which could be explained by a more usual concern about the risk of pathogen transmission (particularly during highly resistant bacteria outbreaks).

At M0 and M3 and in the analysis restricted to high-risk HCWs and accounting for adherence to PPE measures, being a current smoker reduced the risk of laboratory-confirmed SARS-CoV-2 infection (OR 0.36, 95% CI [0.21; 0.63]). An increasing number of studies have reported this reduced risk of SARS-CoV-2 infection in current smokers in different contexts (cross-sectional studies in the general population; cross-sectional, case–control or control studies in different populations)^19–24^. Our results from a large multicenter prospective study in a young population of HCWs with 26.9% current smokers support the role of use of tobacco substances as protective against SARS-CoV-2 infection, which may act through the nicotine pathway^25–28^. This result should not encourage smoking to limit the risk of COVID-19; indeed, 78,000 deaths per year are due to smoking in France^29^. However, the nicotinic hypothesis is of interest, even in the era of an anti-SARS-CoV-2 vaccine, and is being investigating for prevention of COVID-19 (NCT04583410).

The professional category was not a risk factor for laboratory-confirmed SARS-CoV-2 infection, even if medical students exhibited high prevalence as compared with senior physicians (23.5%, 95% CI [20.8–26.4] vs 14.2% [12.2–16.5]) (Table 2). This result may be explained by an insufficient practice or experience with PPE recommendations in medical students or confounded by age. Similarly, reporting high compliance with PPE was not protective (Table 4 and Supplementary Table 1). However, reported adherence to PPE was very good in the SEROCOV population. Regardless, several studies highlighted greater prevalence of SARS-CoV-2 positive serology in HCWs reporting not systematically wearing PPEs in general or covering the face during clinical encounters than in HCWs fully compliant (15.8% vs 4.3%, *p* = 0.07, and 9% vs 6% *p* = 0.012)^5,6,14^. The questionnaire on PPE compliance completed only at the end of follow-up in our study may explain in part this discrepancy due to a potential memory bias in addition to social desirability bias.

The strengths of the study are the prospective design, the recruitment in the early phase of the first epidemic wave; the multicenter, multi-department and multi-professional recruitment of the study; the large sample size; the 3-point serology testing; the centralization of serology assays; and the criteria retained for laboratory-confirmed SARS-CoV-2 infection (rather than seroprevalence alone, which is exposed to negativation). As limitations, first, 11.7% HCWs were lost to follow-up at M3, but missing serology results were handled by multiple imputations. Second, national and center-driven recommandations concerning PPE may have evolved along the study period, while compliance with PPE and standard recommendations were queried only at the end of the study and not weekly, as for the clinical signs, with therefore a potential risk of memory bias and social desirability bias. Third, we did not adjust for clustering by site, which was not possible due to the limited number of centers. Fourth, we did not collect personal risk factors (social and environmental data) as we were focused on professional one’s. This deliberate choice was important in the context of the first wave, to promote adhesion of HCW’s to the study. Finally, the serology assay we used (targeting the viral nucleoprotein) is less sensitive than anti-spike assays^8,12^. Therefore, the reported incidence rate of documented SARS-CoV-2 infection in the SEROCOV study may be slightly underestimated. However, we accounted for this issue in the comparison with seroprevalence data available in the general population.

In summary, in this study of frontline HCWs in Paris, France, we report a 14.6% SARS-CoV-2 cumulative incidence rate at the end of the first COVID-19 wave, with a seroprevalence in May 2020 significantly higher than in the general population. The study of risk factors for laboratory-confirmed SARS-CoV-2 infection argues for a significant part of professional (together with household) contaminations and highlights smoker status as an independent protective factor. At the era of anti-SARS-CoV2 vaccination, our results represent an argument to include HCWs in the first-line target population.

## Supplementary Information

Supplementary Tables.

Supplementary Information 2.

## Data Availability

Data collected for the study, including individual participant data and a data dictionary defining each field in the set, will be made available to others under request. The study protocol and statistical analysis plan will be available on request on an URL of the URC Pitié Salpêtrière Charles Foix.
