# Supplementary material for: Cumulative incidence of SARS-CoV-2 infection and associated risk factors among frontline health care workers in Paris: the SEROCOV cohort study
Source: Sci Rep. 2022 May 4;12:7211. doi: 10.1038/s41598-022-10945-y (PMC9068621; doi:10.1038/s41598-022-10945-y)
Supplement: Supplementary file 1 — Supplementary Tables. [file 41598_2022_10945_MOESM1_ESM.docx]

**Supplementary materials**

**Supplementary table 1**: distribution of the population studied, according to the hospital and department of work

|  |  |  |
| --- | --- | --- |
| *Hospital* | Paris-APHP - Bichat | 172 (15.21%) |
|  | Paris-APHP - Pitié Salpêtrière | 449 (39.7%) |
|  | Paris-APHP - Saint Antoine | 280 (24.76%) |
|  | Paris-APHP - Tenon | 148 (13.09%) |
|  | Paris-APHP - Trousseau | 82 (7.25%) |
| *Type of Department* | Infectious Diseases | 351 (31.03%) |
|  | Intensive Care Unit | 355 (31.39%) |
|  | Emergency Department | 380 (33.6%) |
|  | Virology laboratory | 45 (3.98%) |
| *Hospital - department* | Paris-APHP - Bichat – Infectious Diseases | 172 (15.21%) |
|  | Paris-APHP - Pitié Salpêtrière Infectious Diseases | 49 (4.33%) |
|  | Paris-APHP - Saint Antoine - Infectious Diseases | 130 (11.49%) |
|  | Paris-APHP - Pitié Salpêtrière Intensive Care Unit | 229 (20.25%) |
|  | Paris-APHP - Saint Antoine - Intensive Care Unit | 50 (4.42%) |
|  | Paris-APHP - Tenon - Intensive Care Unit | 76 (6.72%) |
|  | Paris-APHP - Pitié Salpêtrière  Emergency Department | 126 (11.14%) |
|  | Paris-APHP - Saint Antoine - Emergency Department | 100 (8.84%) |
|  | Paris-APHP - Tenon –  Emergency Department | 72 (6.37%) |
|  | Paris-APHP - Trousseau –  Emergency Department | 82 (7.25%) |
|  | Paris-APHP - Pitié Salpêtrière – Virology laboratory | 45 (3.98%) |

**Supplementary table 2 :** Risk factors (multivariate) of laboratory-confirmed SARS-CoV-2 infection at inclusion, before imputation of missing data

| **Characteristic** | **OR^1^** | **95% CI^1^** | **p-value** |
| --- | --- | --- | --- |
| **Age** | 0.98 | 0.94, 1.01 | 0.20 |
| **Sexe** |  |  | 0.43 |
| Male | — | — |  |
| Female | 0.74 | 0.36, 1.61 |  |
| **Working in a referent hospital for emerging biological risk** |  |  | 0.81 |
| no | — | — |  |
| yes | 1.09 | 0.56, 2.17 |  |
| **Working department** |  |  | **<0.001** |
| Emergency department | — | — |  |
| Infectious diseases department | 7.48 | 3.02, 21.5 |  |
| Intensive care unit | 1.53 | 0.55, 4.67 |  |
| **Professional category** |  |  | 0.32 |
| Physician | — | — |  |
| Medical students | 2.13 | 0.50, 8.71 |  |
| Care assistants | 1.10 | 0.34, 3.34 |  |
| nurses | 1.66 | 0.70, 4.23 |  |
| others | 0.52 | 0.11, 1.86 |  |
| **Experience in the department ≥12 months** |  |  | 0.28 |
| no | — | — |  |
| yes | 1.73 | 0.66, 5.34 |  |
| **Experience in the job ≥12 months** |  |  | 0.97 |
| no | — | — |  |
| yes | 1.03 | 0.25, 5.29 |  |
| **Night shift** |  |  | 0.36 |
| no | — | — |  |
| yes | 0.52 | 0.08, 1.91 |  |
| **Public transportation use** |  |  | >0.99 |
| No | — | — |  |
| Yes | 1.00 | 0.51, 1.94 |  |
| **Smoking status** |  |  | **0.012** |
| Past or no smoker | — | — |  |
| Current smoker | 0.30 | 0.09, 0.79 |  |
| ^1^OR = Odds Ratio, CI = Confidence Interval | | | |

**Table 3:** Risk factors (multivariate) of laboratory-confirmed SARS-CoV-2 infection at M3, before imputation of missing data

| **Characteristic** | **OR^1^** | **95% CI^1^** | **p-value** |
| --- | --- | --- | --- |
| **Age** | 0.99 | 0.97, 1.01 | 0.42 |
| **Sexe** |  |  | 0.40 |
| Male | — | — |  |
| Female | 1.23 | 0.76, 2.05 |  |
| **Working in a referent hospital for**  **Emerging biological risk** |  |  | 0.34 |
| No | — | — |  |
| Yes | 1.23 | 0.80, 1.90 |  |
| **Working Department** |  |  | **0.003** |
| Virology laboratory | — | — |  |
| Infectious diseases department | 4.15 | 1.11, 27.2 |  |
| Intensive care unit | 1.85 | 0.47, 12.4 |  |
| Emergency department | 4.01 | 1.03, 26.8 |  |
| **Professional category** |  |  | 0.19 |
| Senior Physician | — | — |  |
| Medical students | 2.40 | 0.91, 6.21 |  |
| Care assistants | 0.93 | 0.47, 1.84 |  |
| Nurses | 1.34 | 0.76, 2.41 |  |
| Others | 0.73 | 0.32, 1.57 |  |
| Experience in the department ≥12 months |  |  | 0.31 |
| no | — | — |  |
| yes | 1.39 | 0.74, 2.76 |  |
| Experience in the job ≥12 months |  |  | 0.72 |
| no | — | — |  |
| yes | 0.86 | 0.37, 2.05 |  |
| **Night shift** |  |  | 0.94 |
| no | — | — |  |
| yes | 0.98 | 0.49, 1.83 |  |
| **Public transportation use** |  |  | 0.49 |
| No | — | — |  |
| yes | 1.15 | 0.77, 1.73 |  |
| **Smoking status** |  |  | **<0.001** |
| Past or no smoker | — | — |  |
| Current smoker | 0.38 | 0.21, 0.66 |  |
| ^1^OR = Odds Ratio, CI = Confidence Interval | | | |

**Supplementary table 4 :** Risk factors (multivariate) of laboratory-confirmed SARS-CoV-2 infection at month 3 in high-risk healthcare workers, accounting for adherence to personal protective equipment recommendations, before imputation of missing data.

| **Characteristic** | **OR^1^** | **95% CI^1^** | **p-value** |
| --- | --- | --- | --- |
| **Age** | 0.99 | 0.96, 1.02 | 0.52 |
| **Sexe** |  |  | 0.87 |
| Male | — | — |  |
| Female | 1.05 | 0.58, 1.99 |  |
| Working in a referent hospital  for emerging biological risk |  |  | 0.57 |
| no | — | — |  |
| yes | 1.17 | 0.68, 2.01 |  |
| **Working department** |  |  | **0.006** |
| Intensive care unit | — | — |  |
| Infectious diseases | 3.05 | 1.53, 6.14 |  |
| Emergency department | 1.88 | 0.96, 3.72 |  |
| **Professional category** |  |  | 0.41 |
| Senior physician | — | — |  |
| Medical students | 0.84 | 0.19, 3.22 |  |
| Care assistants | 1.22 | 0.49, 3.04 |  |
| Nurses | 1.68 | 0.82, 3.60 |  |
| **Experience in the department ≥12 months** |  |  | 0.79 |
| no | — | — |  |
| yes | 0.91 | 0.45, 1.95 |  |
| **Experience in the job ≥12 months** |  |  | 0.44 |
| no | — | — |  |
| yes | 1.52 | 0.53, 4.62 |  |
| **Night shift** |  |  | 0.89 |
| no | — | — |  |
| yes | 0.95 | 0.44, 1.94 |  |
| **Public transportation use** |  |  | 0.81 |
| no | — | — |  |
| yes | 1.07 | 0.64, 1.76 |  |
| **Smoking status** |  |  | **0.004** |
| Past or no smoker | — | — |  |
| Current smoker | 0.40 | 0.20, 0.76 |  |
| **Wear a surgical mask (0 = never 5 = systematically)** |  |  | 0.35 |
| 4-5 | — | — |  |
| < 4 | 0.61 | 0.17, 1.66 |  |
| **Wear an N95 mask to take nasopharyngeal swabs** |  |  | 0.91 |
| 4-5 | — | — |  |
| < 4 | 1.04 | 0.52, 1.98 |  |
| **Wear an N95 mask to handle a confirmed COVID-19 case** |  |  | 0.39 |
| 4-5 | — | — |  |
| < 4 | 1.31 | 0.71, 2.40 |  |
| **If wearing a mask (surgical or N95), change every 4 hr** |  |  | 0.10 |
| 4-5 | — | — |  |
| < 4 | 1.59 | 0.92, 2.74 |  |
| ^1^OR = Odds Ratio, CI = Confidence Interval | | | |

**Supplementary table 5 :** adherence to PPE recommendations at M0 using a Likert scale from 0-5

|  | | **Serological status M0** | | | |
| --- | --- | --- | --- | --- | --- |
| **label** | **variable** | **Negative** | **Positive** | **Total** | **NA** |
| **Wear a surgical mask (0 = never 5 = systematically)** | 0 | 7 (0.79%) | 0 (0%) | 7 (0.75%) | 0 |
|  | 1 | 4 (0.45%) | 0 (0%) | 4 (0.43%) | 0 |
|  | 2 | 6 (0.68%) | 2 (4.26%) | 8 (0.86%) | 0 |
|  | 3 | 41 (4.62%) | 5 (10.64%) | 46 (4.93%) | 0 |
|  | 4 | 170 (19.17%) | 13 (27.66%) | 183 (19.59%) | 0 |
|  | 5 | 659 (74.3%) | 27 (57.45%) | 686 (73.45%) | 1 |
|  | Total | 887 (94.97%) | 47 (5.03%) | 934 (100%) | 2 |
|  | NA | 112 | 14 | 127 | 1 |
| **Wear a surgical mask (0 = never 5 = systematically)** | 4-5 | 829 (93.46%) | 40 (85.11%) | 869 (93.04%) | 1 |
|  | < 4 | 58 (6.54%) | 7 (14.89%) | 65 (6.96%) | 0 |
|  | Total | 887 (94.97%) | 47 (5.03%) | 934 (100%) | 2 |
|  | NA | 112 | 14 | 127 | 1 |
| **Wear an N95 mask to take nasopharyngeal swabs** | 0 | 71 (10.57%) | 2 (5.88%) | 73 (10.34%) | 0 |
|  | 1 | 7 (1.04%) | 0 (0%) | 7 (0.99%) | 0 |
|  | 2 | 22 (3.27%) | 2 (5.88%) | 24 (3.4%) | 0 |
|  | 3 | 51 (7.59%) | 2 (5.88%) | 53 (7.51%) | 0 |
|  | 4 | 76 (11.31%) | 8 (23.53%) | 84 (11.9%) | 0 |
|  | 5 | 445 (66.22%) | 20 (58.82%) | 465 (65.86%) | 0 |
|  | Total | 672 (95.18%) | 34 (4.82%) | 706 (100%) | 2 |
|  | NA | 327 | 27 | 356 | 2 |
| **Wear an N95 mask to take nasopharyngeal swabs** | 4-5 | 521 (77.53%) | 28 (82.35%) | 549 (77.76%) | 0 |
|  | < 4 | 151 (22.47%) | 6 (17.65%) | 157 (22.24%) | 0 |
|  | Total | 672 (95.18%) | 34 (4.82%) | 706 (100%) | 2 |
|  | NA | 327 | 27 | 356 | 2 |
| **Wear an N95 mask to handle a confirmed COVID-19 case** | 0 | 62 (7.95%) | 5 (11.63%) | 67 (8.14%) | 0 |
|  | 1 | 31 (3.97%) | 2 (4.65%) | 33 (4.01%) | 0 |
|  | 2 | 38 (4.87%) | 5 (11.63%) | 43 (5.22%) | 0 |
|  | 3 | 71 (9.1%) | 7 (16.28%) | 78 (9.48%) | 0 |
|  | 4 | 84 (10.77%) | 1 (2.33%) | 85 (10.33%) | 0 |
|  | 5 | 494 (63.33%) | 23 (53.49%) | 517 (62.82%) | 1 |
|  | Total | 780 (94.78%) | 43 (5.22%) | 823 (100%) | 2 |
|  | NA | 219 | 18 | 238 | 1 |
| **Wear an N95 mask to handle a confirmed COVID-19 case** | 4-5 | 578 (74.1%) | 24 (55.81%) | 602 (73.15%) | 1 |
|  | < 4 | 202 (25.9%) | 19 (44.19%) | 221 (26.85%) | 0 |
|  | Total | 780 (94.78%) | 43 (5.22%) | 823 (100%) | 2 |
|  | NA | 219 | 18 | 238 | 1 |
| **If wearing a mask (surgical or N95), change every 4 hr** | 0 | 63 (7.16%) | 2 (4.26%) | 65 (7.01%) | 0 |
|  | 1 | 39 (4.43%) | 4 (8.51%) | 43 (4.64%) | 0 |
|  | 2 | 69 (7.84%) | 9 (19.15%) | 78 (8.41%) | 0 |
|  | 3 | 139 (15.8%) | 10 (21.28%) | 149 (16.07%) | 0 |
|  | 4 | 183 (20.8%) | 5 (10.64%) | 188 (20.28%) | 0 |
|  | 5 | 387 (43.98%) | 17 (36.17%) | 404 (43.58%) | 0 |
|  | Total | 880 (94.93%) | 47 (5.07%) | 927 (100%) | 2 |
|  | NA | 119 | 14 | 135 | 2 |
| **If wearing a mask (surgical or N95), change every 4 hr** | 4-5 | 570 (64.77%) | 22 (46.81%) | 592 (63.86%) | 0 |
|  | < 4 | 310 (35.23%) | 25 (53.19%) | 335 (36.14%) | 0 |
|  | Total | 880 (94.93%) | 47 (5.07%) | 927 (100%) | 2 |
|  | NA | 119 | 14 | 135 | 2 |

**Supplementary material 11 : power calculation**

*The purpose of the study was to estimate a proportion of HCW with documented SARS-CoV2 infections at M3 (at the end of the first wave of the pandemics) in a range of services and hospitals with different characteristics. All eligible HCW of participating services were to be solicited, and all those who agree to participate would be included. We included hospitals with different characteristics (referent hospital for emerging biological risk or not, hospital for adults or for children, large or small hospitals), and the main departments involved in the care of COVID patients at the very beginning of the pandemics. We did not have data on the expected proportion of documented SARS CoV2 infections that we will observe. Thus we estimated the precision that can be obtained according to different scenarii (exact confidence intervals, N: numbers included) if we include 400, 600, 800 or 1000 HCW (see table below), and planned to include 1000 HCW to have a good precision on the estimation. We did not use a proportional allocation in each department, and solicited all eligible HCW.*

| Proportion of HCW with documented SARS-CoV2 infection at M3 | 95% confidence interval of the  proportion if N=400 | 95% confidence interval of the proportion if N=600 | 95% confidence interval of the proportion if N=800 | 95% confidence interval of the proportion if N=1000 |
| --- | --- | --- | --- | --- |
| 5 % | 3.1% ; 7.6% | 3.4% ; 7.1% | 3.6% ; 6.7% | 3.7% ; 6.5% |
| 10 % | 7.2% ; 13.4% | 7.7% ; 12.7% | 8.0% ; 12.3% | 8.2% ; 12.0% |
| 20 % | 16.2% ; 24.3% | 16.9% ; 23.4% | 17.3% ; 22.9% | 17.6% ; 22.6% |
| 50 % | 45.0% ; 55.0% | 45.9% ; 54.1% | 46.5% ; 53.5% | 46.9% ; 53.1% |
| 70 % | 65.2% ; 74.5% | 66.2% ; 73.6% | 66.7% ; 73.2% | 67.1% ; 72.8% |
