# Supplementary material for: Cumulative incidence of SARS-CoV-2 infection and associated risk factors among frontline health care workers in Paris: the SEROCOV cohort study
Source: Sci Rep. 2022 May 4;12:7211. doi: 10.1038/s41598-022-10945-y (PMC9068621; doi:10.1038/s41598-022-10945-y)
Supplement: Supplementary file 2 — Supplementary Information 2. [file 41598_2022_10945_MOESM2_ESM.pdf]

Cahier vierge
Protocole
SEROCOV
Version 47

19/05/2020 11:03

Visite d'inclusion

Identification	
SEROCOV	
Nom (première lettre)	|__|
Prénom (première lettre)	|__|
Numéro du centre	|__|__|__|
Nom de centre	....
Référence du patient	....
Nom de l'investigateur	....
Age	|__|__|__|__|__|__|
Sexe	◯ Masculin ◯ Féminin
Date de signature de consentement	|__|__|/|__|__|/|__|__|__|__|
Date d'inclusion	|__|__|/|__|__|/|__|__|__|__|

Critères d'inclusion

1. Personnel médical ou paramédical permanent dans l'un des services participants. On entend ici par permanents les personnels du service, qu'ils soient sur des postes pérennes ou non (donc y compris internes, chefs de cliniques...) par opposition aux personnels d'autres services qui y prennent des gardes ponctuellement.	◯ Oui ◯ Non
2. Consentement écrit	◯ Oui ◯ Non
3. Personnel bénéficiaire d'un régime de sécurité sociale	◯ Oui ◯ Non

Si l'un des items ci-dessus est à « Non », le participant ne peut être inclus dans l'étude

Critères de non inclusion

1. Personnel sans activité dans le service concerné pendant la période d'inclusion	◯ Oui ◯ Non
2. Personnel non permanent (prise occasionnelle de gardes, personnel du pool de l'hôpital, intérimaires), personnel administratif, personnel technique des services participants	◯ Oui ◯ Non
3. Personnel faisant l'objet d'une mesure de protection de justice	◯ Oui ◯ Non

Si l'un des items ci-dessus est à « Oui », le participant ne peut être inclus dans l'étude

Visite d'inclusion (Suite)

Données sociodémographiques

PM	○ Interne/Externe<br>○ CCA/AHU<br>○ PA<br>○ PHC<br>○ PH<br>○ MCU-PH<br>○ PUPH
PNM	○ AS<br>○ IDE<br>○ Cadre<br>○ KINE<br>○ Autre profession PNM
Préciser	....
Horaires de travail	○ Jour<br>○ Garde<br>○ Nuit<br>○ Mixte
Nombre de mois d'exercice dans le service	|__|__|__|
Nombre de mois d'exercice dans le métier	|__|__|__|
Prélèvement sanguin veineux	○ Fait ○ Non fait
Remise d'un carnet d'auto-surveillance hebdomadaire	○ Oui ○ Non

Visite intermédiaire

Visite Intermédiaire	
Durant sa participation à l'étude, le personnel soignant a-t-il développé<br>des symptômes significatifs (= entrainant une atteinte/ un changement<br>de sa qualité de vie) de syndrome viral clinique aigu ?	◯ Oui ◯ Non
1 - A-t-il contacté l'investigateur ?	
1 - A-t-il contacté l'investigateur ?	◯ Oui ◯ Non
A quelle date	|__|__|/|__|__|/|__|__|__|__|
2 - Préciser le type de symptômes	
Signes généraux	☐ Fièvre (> 38°C)<br>☐ Courbatures<br>☐ Frissons<br>☐ Sueurs importantes<br>☐ Céphalées<br>☐ Perte du goût (agueusie)<br>☐ Perte de l'odorat (anosmie)
Respiratoires	☐ Toux<br>☐ Douleur de gorge<br>☐ Expectoration<br>☐ Dyspnée
Digestifs	☐ Nausées<br>☐ Vomissements<br>☐ Diarrhée<br>☐ Douleur abdominale
3 - Préciser la réalisation du prélèvement intermédiaire<br>(10 jours après le début des symptômes)	
Prélèvement sanguin veineux	◯ Fait ◯ Non Fait
Si fait, date	|__|__|/|__|__|/|__|__|__|__|
Diagnostic posé	....
Prélèvement respiratoire virocult réalisé	◯ Fait ◯ Non Fait
Si fait, date	|__|__|/|__|__|/|__|__|__|__|
Résultat	....

Visite de suivi M1

Visite de suivi M1	
Date	|__|__|/|__|__|/|__|__|__|__|
Prélèvement sanguin veineux	○ Fait ○ Non Fait
Si fait, date	|__|__|/|__|__|/|__|__|__|__|
Raison	....
Avez-vous eux des symptômes avant la visite d'inclusion (J1) ?	○ Oui ○ Non
Quels ont été vos symptômes ?	....

Visite de fin d'étude M3

Visite de fin d'étude	
Date	|__|__|/|__|__|/|__|__|__|__|
Pendant les 3 derniers mois, avez-vous été absents ?	○ Oui ○ Non
Si oui, combien de jours (>= 7 jours) :	|__|__| Jours
Prélèvement sanguin veineux :	○ Fait ○ Non Fait
Récupération du carnet d'auto-surveillance :	○ Oui ○ Non
Carnet perdu	○ Oui ○ Non
Collecte des signes en rétrospectif	○ Oui ○ Non
**Préciser le type de symptômes :**	
Signes généraux	☐ Fièvre (> 38°C)<br>☐ Courbatures<br>☐ Frissons<br>☐ Sueurs importantes<br>☐ Céphalées
Respiratoires	☐ Toux<br>☐ Douleur de gorge<br>☐ Expectoration<br>☐ Dyspnée
Digestifs	☐ Nausées<br>☐ Vomissements<br>☐ Diarrhée<br>☐ Douleur abdominale
**Application des mesures de Protection recommandées :**	
En moyenne, durant les 3 mois de l'étude, sur une échelle de 0 à 5, comment qualifieriez-vous votre pratique concernant les mesures de protection individuelles : (0= jamais à 5= systématiquement)	
Port d'un masque chirurgical	|__|(0-5)
Port d'un masque FFP2 pour réaliser les prélèvements naso-pharyngés (tout patient)	|__|(0-5)
Port d'un masque FFP2 pour la prise en charge d'un cas confirmé COVID-19	|__|(0-5)
Si port d'un masque (chirurgical ou FFP2 : changement toutes les 4h	|__|(0-5)
Friction hydro-alcoolique avant et après tout contact avec un patient	|__|(0-5)
Lavage des mains avant et après tout contact avec un patient	|__|(0-5)

Carnet d'auto-évaluation

Statut tabagique

Statut tabagique : ○ Fumeur actif ○ Fumeur Passif ○ Non fumeur

Semaine1	
Date d'évaluation	|__|__|/|__|__|/|__|__|__|__|
Si vous ressentez, de manière significative (=entrainant une atteinte/un changement de votre qualité de vie), un des symptômes suivants, merci de contacter le PM référent. Merci de répondre aux 8 questions suivantes.	
1-Au cours de la semaine précédente, avez-vous été absent ?	○ Oui ○ Non
Si oui, combien de jours	|__|__|__|__|__|__|
2 - Au cours de la semaine, avez-vous eu :	
- de la fièvre (>38°C) :	○ Oui ○ Non
Si oui, combien de jours	|__|__|__|__|__|__|
- des courbatures :	○ Oui ○ Non
Si oui, combien de jours	|__|__|__|__|__|__|
- des frissons :	○ Oui ○ Non
Si oui, combien de jours	|__|__|__|__|__|__|
- des sueurs importantes :	○ Oui ○ Non
Si oui, combien de jours	|__|__|__|__|__|__|
- des céphalées:	○ Oui ○ Non
Si oui, combien de jours	|__|__|__|__|__|__|
-perte de l'odorat (anosmie)	○ Oui ○ Non
Si oui, combien de jours	|__|__|__|__|__|__|
-perte du goût (agueusie)	○ Oui ○ Non
Si oui, combien de jours	|__|__|__|__|__|__|
3 - Si vous avez répondu OUI à une des questions précédentes, avez-vous eu :	
- signes respiratoires : toux, douleur de gorge, expectoration, dyspnée	○ Oui ○ Non
- signes digestifs : nausées, vomissements, diarrhée, douleur abdominale	○ Oui ○ Non
4 - Si vous avez répondu OUI à l'une des questions précédentes (2 ou 3),<br>avez-vous dû être arrêté pour ces symptômes ?	○ Oui ○ Non
Si oui, combien de jours	|__|__|__|__|__|__|
5 - Si vous avez répondu OUI à l'une des questions précédentes<br>avez-vous dû être hospitalisé pour ces symptômes ?	○ Oui ○ Non
Si oui, combien de jours	|__|__|__|__|__|__|
6 - Un diagnostic a-t-il été posé ?	○ Oui ○ Non
Si oui, lequel ?	....
7 - Avez-vous été en contact avec un cas documenté ?	○ Oui ○ Non
8- Quel est votre moyen de transport régulier pour venir au travail ?	☐ Véhicule personnel<br>☐ Transport en commun<br>☐ A pied<br>☐ Vélo/trottinette<br>☐ Autres
Préciser	....
Préciser	○ Personnel ○ Libre-service

Semaine2	
Date d'évaluation	|__|__|/|__|__|/|__|__|__|__|
Si vous ressentez, de manière significative (=entrainant une atteinte/un changement de votre qualité de vie), un des symptômes suivants,merci de contacter le PM référent. Merci de répondre aux 8 questions suivantes.	
1- Au cours de la semaine précédente, avez-vous été absent?	○ Oui ○ Non
Si oui, combien de jours	|__|__|__|__|__|__|
2 - Au cours de la semaine, avez-vous eu :	
- de la fièvre (>38°C) :	○ Oui ○ Non
Si oui, combien de jours	|__|__|__|__|__|__|
- des courbatures :	○ Oui ○ Non
Si oui, combien de jours	|__|__|__|__|__|__|
- des frissons :	○ Oui ○ Non
Si oui, combien de jours	|__|__|__|__|__|__|
- des sueurs importantes :	○ Oui ○ Non
Si oui, combien de jours	|__|__|__|__|__|__|
- des céphalées:	○ Oui ○ Non
Si oui, combien de jours	|__|__|__|__|__|__|
-perte de l'odorat (anosmie)	○ Oui ○ Non
Si oui, combien de jours	|__|__|__|__|__|__|
-perte du goût (agueusie)	○ Oui ○ Non
Si oui, combien de jours	|__|__|__|__|__|__|
3 - Si vous avez répondu OUI à une des questions précédentes, avez-vous eu :	
- signes respiratoires : toux, douleur de gorge, expectoration, dyspnée	○ Oui ○ Non
- signes digestifs : nausées, vomissements, diarrhée, douleur abdominale	○ Oui ○ Non
4 - Si vous avez répondu OUI à l'une des questions précédentes (2 ou 3),<br>avez-vous dû être arrêté pour ces symptômes ?	○ Oui ○ Non
Si oui, combien de jours	|__|__|__|__|__|__|
5 - Si vous avez répondu OUI à l'une des questions précédentes<br>avez-vous dû être hospitalisé pour ces symptômes ?	○ Oui ○ Non
Si oui, combien de jours	|__|__|__|__|__|__|
6 - Un diagnostic a-t-il été posé ?	○ Oui ○ Non
Si oui, lequel ?	....
7 - Avez-vous été en contact avec un cas documenté ?	○ Oui ○ Non
8- Quel est votre moyen de transport régulier pour venir au travail ?	☐ Véhicule personnel<br>☐ Transport en commun<br>☐ A pied<br>☐ Vélo/trottinette<br>☐ Autres
Préciser	....
Préciser	○ Personnel ○ Libre-service

Semaine3	
Date d'évaluation	|__|__|/|__|__|/|__|__|__|__|
Si vous ressentez, de manière significative (=entrainant une atteinte/un changement de votre qualité de vie), un des symptômes suivants,merci de contacter le PM référent. Merci de répondre aux 8 questions suivantes.	
1- Au cours de la semaine précédente, avez-vous été absent?	○ Oui ○ Non
Si oui, combien de jours	|__|__|__|__|__|__|
2 - Au cours de la semaine, avez-vous eu :	
- de la fièvre (>38°C) :	○ Oui ○ Non
Si oui, combien de jours	|__|__|__|__|__|__|
- des courbatures :	○ Oui ○ Non
Si oui, combien de jours	|__|__|__|__|__|__|
- des frissons :	○ Oui ○ Non
Si oui, combien de jours	|__|__|__|__|__|__|
- des sueurs importantes :	○ Oui ○ Non
Si oui, combien de jours	|__|__|__|__|__|__|
- des céphalées:	○ Oui ○ Non
Si oui, combien de jours	|__|__|__|__|__|__|
-perte de l'odorat (anosmie)	○ Oui ○ Non
Si oui, combien de jours	|__|__|__|__|__|__|
-perte du goût (agueusie)	○ Oui ○ Non
Si oui, combien de jours	|__|__|__|__|__|__|
3 - Si vous avez répondu OUI à une des questions précédentes, avez-vous eu :	
- signes respiratoires : toux, douleur de gorge, expectoration, dyspnée	○ Oui ○ Non
- signes digestifs : nausées, vomissements, diarrhée, douleur abdominale	○ Oui ○ Non
4 - Si vous avez répondu OUI à l'une des questions précédentes (2 ou 3),<br>avez-vous dû être arrêté pour ces symptômes ?	○ Oui ○ Non
Si oui, combien de jours	|__|__|__|__|__|__|
5 - Si vous avez répondu OUI à l'une des questions précédentes<br>avez-vous dû être hospitalisé pour ces symptômes ?	○ Oui ○ Non
Si oui, combien de jours	|__|__|__|__|__|__|
6 - Un diagnostic a-t-il été posé ?	○ Oui ○ Non
Si oui, lequel ?	....
7 - Avez-vous été en contact avec un cas documenté ?	○ Oui ○ Non
8- Quel est votre moyen de transport régulier pour venir au travail ?	☐ Véhicule personnel<br>☐ Transport en commun<br>☐ A pied<br>☐ Vélo/trottinette<br>☐ Autres
Préciser	....
Préciser	○ Personnel ○ Libre-service

Semaine4	
Date d'évaluation	|__|__|/|__|__|/|__|__|__|__|
Si vous ressentez, de manière significative (=entrainant une atteinte/un changement de votre qualité de vie), un des symptômes suivants, merci de contacter le PM référent. Merci de répondre aux 8 questions suivantes.	
1-Au cours de la semaine précédente, avez-vous été absent?	○ Oui ○ Non
Si oui, combien de jours	|__|__|__|__|__|__|
2 - Au cours de la semaine, avez-vous eu :	
- de la fièvre (>38°C) :	○ Oui ○ Non
Si oui, combien de jours	|__|__|__|__|__|__|
- des courbatures :	○ Oui ○ Non
Si oui, combien de jours	|__|__|__|__|__|__|
- des frissons :	○ Oui ○ Non
Si oui, combien de jours	|__|__|__|__|__|__|
- des sueurs importantes :	○ Oui ○ Non
Si oui, combien de jours	|__|__|__|__|__|__|
- des céphalées:	○ Oui ○ Non
Si oui, combien de jours	|__|__|__|__|__|__|
-perte de l'odorat (anosmie)	○ Oui ○ Non
Si oui, combien de jours	|__|__|__|__|__|__|
-perte du goût (agueusie)	○ Oui ○ Non
Si oui, combien de jours	|__|__|__|__|__|__|
3 - Si vous avez répondu OUI à une des questions précédentes, avez-vous eu :	
- signes respiratoires : toux, douleur de gorge, expectoration, dyspnée	○ Oui ○ Non
- signes digestifs : nausées, vomissements, diarrhée, douleur abdominale	○ Oui ○ Non
4 - Si vous avez répondu OUI à l'une des questions précédentes (2 ou 3),<br>avez-vous dû être arrêté pour ces symptômes ?	○ Oui ○ Non
Si oui, combien de jours	|__|__|__|__|__|__|
5 - Si vous avez répondu OUI à l'une des questions précédentes<br>avez-vous dû être hospitalisé pour ces symptômes ?	○ Oui ○ Non
Si oui, combien de jours	|__|__|__|__|__|__|
6 - Un diagnostic a-t-il été posé ?	○ Oui ○ Non
Si oui, lequel ?	....
7 - Avez-vous été en contact avec un cas documenté ?	○ Oui ○ Non
8- Quel est votre moyen de transport régulier pour venir au travail ?	☐ Véhicule personnel<br>☐ Transport en commun<br>☐ A pied<br>☐ Vélo/trottinette<br>☐ Autres
Préciser	....
Préciser	○ Personnel ○ Libre-service

Semaine5	
Date d'évaluation	|__|__|/|__|__|/|__|__|__|__|
Si vous ressentez, de manière significative (=entrainant une atteinte/un changement de votre qualité de vie), un des symptômes suivants,merci de contacter le PM référent. Merci de répondre aux 8 questions suivantes.	
1- Au cours de la semaine précédente, avez-vous été absent?	○ Oui ○ Non
Si oui, combien de jours	|__|__|__|__|__|__|
2 - Au cours de la semaine, avez-vous eu :	
- de la fièvre (>38°C) :	○ Oui ○ Non
Si oui, combien de jours	|__|__|__|__|__|__|
- des courbatures :	○ Oui ○ Non
Si oui, combien de jours	|__|__|__|__|__|__|
- des frissons :	○ Oui ○ Non
Si oui, combien de jours	|__|__|__|__|__|__|
- des sueurs importantes :	○ Oui ○ Non
Si oui, combien de jours	|__|__|__|__|__|__|
- des céphalées:	○ Oui ○ Non
Si oui, combien de jours	|__|__|__|__|__|__|
-perte de l'odorat (anosmie)	○ Oui ○ Non
Si oui, combien de jours	|__|__|__|__|__|__|
-perte du goût (agueusie)	○ Oui ○ Non
Si oui, combien de jours	|__|__|__|__|__|__|
3 - Si vous avez répondu OUI à une des questions précédentes, avez-vous eu :	
- signes respiratoires : toux, douleur de gorge, expectoration, dyspnée	○ Oui ○ Non
- signes digestifs : nausées, vomissements, diarrhée, douleur abdominale	○ Oui ○ Non
4 - Si vous avez répondu OUI à l'une des questions précédentes (2 ou 3),<br>avez-vous dû être arrêté pour ces symptômes ?	○ Oui ○ Non
Si oui, combien de jours	|__|__|__|__|__|__|
5 - Si vous avez répondu OUI à l'une des questions précédentes<br>avez-vous dû être hospitalisé pour ces symptômes ?	○ Oui ○ Non
Si oui, combien de jours	|__|__|__|__|__|__|
6 - Un diagnostic a-t-il été posé ?	○ Oui ○ Non
Si oui, lequel ?	....
7 - Avez-vous été en contact avec un cas documenté ?	○ Oui ○ Non
8- Quel est votre moyen de transport régulier pour venir au travail ?	☐ Véhicule personnel<br>☐ Transport en commun<br>☐ A pied<br>☐ Vélo/trottinette<br>☐ Autres
Préciser	....
Préciser	○ Personnel ○ Libre-service

**Semaine6**	
Date d'évaluation	|__|__|/|__|__|/|__|__|__|__|
Si vous ressentez, de manière significative (=entrainant une atteinte/un changement de votre qualité de vie), un des symptômes suivants,merci de contacter le PM référent. Merci de répondre aux 8 questions suivantes.	
1-Au cours de la semaine précédente, avez-vous été absent?	○ Oui ○ Non
Si oui, combien de jours	|__|__|__|__|__|__|
2 - Au cours de la semaine, avez-vous eu :	
- de la fièvre (>38°C) :	○ Oui ○ Non
Si oui, combien de jours	|__|__|__|__|__|__|
- des courbatures :	○ Oui ○ Non
Si oui, combien de jours	|__|__|__|__|__|__|
- des frissons :	○ Oui ○ Non
Si oui, combien de jours	|__|__|__|__|__|__|
- des sueurs importantes :	○ Oui ○ Non
Si oui, combien de jours	|__|__|__|__|__|__|
- des céphalées:	○ Oui ○ Non
Si oui, combien de jours	|__|__|__|__|__|__|
-perte de l'odorat (anosmie)	○ Oui ○ Non
Si oui, combien de jours	|__|__|__|__|__|__|
-perte du goût (agueusie)	○ Oui ○ Non
Si oui, combien de jours	|__|__|__|__|__|__|
3 - Si vous avez répondu OUI à une des questions précédentes, avez-vous eu :	
- signes respiratoires : toux, douleur de gorge, expectoration, dyspnée	○ Oui ○ Non
- signes digestifs : nausées, vomissements, diarrhée, douleur abdominale	○ Oui ○ Non
4 - Si vous avez répondu OUI à l'une des questions précédentes (2 ou 3),<br>avez-vous dû être arrêté pour ces symptômes ?	○ Oui ○ Non
Si oui, combien de jours	|__|__|__|__|__|__|
5 - Si vous avez répondu OUI à l'une des questions précédentes<br>avez-vous dû être hospitalisé pour ces symptômes ?	○ Oui ○ Non
Si oui, combien de jours	|__|__|__|__|__|__|
6 - Un diagnostic a-t-il été posé ?	○ Oui ○ Non
Si oui, lequel ?	....
7 - Avez-vous été en contact avec un cas documenté ?	○ Oui ○ Non
8- Quel est votre moyen de transport régulier pour venir au travail ?	☐ Véhicule personnel<br>☐ Transport en commun<br>☐ A pied<br>☐ Vélo/trottinette<br>☐ Autres
Préciser	....
Préciser	○ Personnel ○ Libre-service

Semaine7	
Date d'évaluation	|__|__|/|__|__|/|__|__|__|__|
Si vous ressentez, de manière significative (=entrainant une atteinte/un changement de votre qualité de vie), un des symptômes suivants,merci de contacter le PM référent. Merci de répondre aux 8 questions suivantes.	
1-Au cours de la semaine précédente, avez-vous été absent?	○ Oui ○ Non
Si oui, combien de jours	|__|__|__|__|__|__|
2 - Au cours de la semaine, avez-vous eu :	
- de la fièvre (>38°C) :	○ Oui ○ Non
Si oui, combien de jours	|__|__|__|__|__|__|
- des courbatures :	○ Oui ○ Non
Si oui, combien de jours	|__|__|__|__|__|__|
- des frissons :	○ Oui ○ Non
Si oui, combien de jours	|__|__|__|__|__|__|
- des sueurs importantes :	○ Oui ○ Non
Si oui, combien de jours	|__|__|__|__|__|__|
- des céphalées:	○ Oui ○ Non
Si oui, combien de jours	|__|__|__|__|__|__|
-perte de l'odorat (anosmie)	○ Oui ○ Non
Si oui, combien de jours	|__|__|__|__|__|__|
-perte du goût (agueusie)	○ Oui ○ Non
Si oui, combien de jours	|__|__|__|__|__|__|
3 - Si vous avez répondu OUI à une des questions précédentes, avez-vous eu :	
- signes respiratoires : toux, douleur de gorge, expectoration, dyspnée	○ Oui ○ Non
- signes digestifs : nausées, vomissements, diarrhée, douleur abdominale	○ Oui ○ Non
4 - Si vous avez répondu OUI à l'une des questions précédentes (2 ou 3),<br>avez-vous dû être arrêté pour ces symptômes ?	○ Oui ○ Non
Si oui, combien de jours	|__|__|__|__|__|__|
5 - Si vous avez répondu OUI à l'une des questions précédentes<br>avez-vous dû être hospitalisé pour ces symptômes ?	○ Oui ○ Non
Si oui, combien de jours	|__|__|__|__|__|__|
6 - Un diagnostic a-t-il été posé ?	○ Oui ○ Non
Si oui, lequel ?	....
7 - Avez-vous été en contact avec un cas documenté ?	○ Oui ○ Non
8- Quel est votre moyen de transport régulier pour venir au travail ?	☐ Véhicule personnel<br>☐ Transport en commun<br>☐ A pied<br>☐ Vélo/trottinette<br>☐ Autres
Préciser	....
Préciser	○ Personnel ○ Libre-service

Semaine8	
Date d'évaluation	|__|__|/|__|__|/|__|__|__|__|
Si vous ressentez, de manière significative (=entrainant une atteinte/un changement de votre qualité de vie), un des symptômes suivants,merci de contacter le PM référent. Merci de répondre aux 8 questions suivantes.	
1-Au cours de la semaine précédente, avez-vous été absent?	○ Oui ○ Non
Si oui, combien de jours	|__|__|__|__|__|__|
2 - Au cours de la semaine, avez-vous eu :	
- de la fièvre (>38°C) :	○ Oui ○ Non
Si oui, combien de jours	|__|__|__|__|__|__|
- des courbatures :	○ Oui ○ Non
Si oui, combien de jours	|__|__|__|__|__|__|
- des frissons :	○ Oui ○ Non
Si oui, combien de jours	|__|__|__|__|__|__|
- des sueurs importantes :	○ Oui ○ Non
Si oui, combien de jours	|__|__|__|__|__|__|
- des céphalées:	○ Oui ○ Non
Si oui, combien de jours	|__|__|__|__|__|__|
-perte de l'odorat (anosmie)	○ Oui ○ Non
Si oui, combien de jours	|__|__|__|__|__|__|
-perte du goût (agueusie)	○ Oui ○ Non
Si oui, combien de jours	|__|__|__|__|__|__|
3 - Si vous avez répondu OUI à une des questions précédentes, avez-vous eu :	
- signes respiratoires : toux, douleur de gorge, expectoration, dyspnée	○ Oui ○ Non
- signes digestifs : nausées, vomissements, diarrhée, douleur abdominale	○ Oui ○ Non
4 - Si vous avez répondu OUI à l'une des questions précédentes (2 ou 3),<br>avez-vous dû être arrêté pour ces symptômes ?	○ Oui ○ Non
Si oui, combien de jours	|__|__|__|__|__|__|
5 - Si vous avez répondu OUI à l'une des questions précédentes<br>avez-vous dû être hospitalisé pour ces symptômes ?	○ Oui ○ Non
Si oui, combien de jours	|__|__|__|__|__|__|
6 - Un diagnostic a-t-il été posé ?	○ Oui ○ Non
Si oui, lequel ?	....
7 - Avez-vous été en contact avec un cas documenté ?	○ Oui ○ Non
8- Quel est votre moyen de transport régulier pour venir au travail ?	☐ Véhicule personnel<br>☐ Transport en commun<br>☐ A pied<br>☐ Vélo/trottinette<br>☐ Autres
Préciser	....
Préciser	○ Personnel ○ Libre-service

Semaine9	
Date d'évaluation	|__|__|/|__|__|/|__|__|__|__|
Si vous ressentez, de manière significative (=entrainant une atteinte/un changement de votre qualité de vie), un des symptômes suivants,merci de contacter le PM référent. Merci de répondre aux 8 questions suivantes.	
1- Au cours de la semaine précédente, avez-vous été absent?	○ Oui ○ Non
Si oui, combien de jours	|__|__|__|__|__|__|
2 - Au cours de la semaine, avez-vous eu :	
- de la fièvre (>38°C) :	○ Oui ○ Non
Si oui, combien de jours	|__|__|__|__|__|__|
- des courbatures :	○ Oui ○ Non
Si oui, combien de jours	|__|__|__|__|__|__|
- des frissons :	○ Oui ○ Non
Si oui, combien de jours	|__|__|__|__|__|__|
- des sueurs importantes :	○ Oui ○ Non
Si oui, combien de jours	|__|__|__|__|__|__|
- des céphalées:	○ Oui ○ Non
Si oui, combien de jours	|__|__|__|__|__|__|
-perte de l'odorat (anosmie)	○ Oui ○ Non
Si oui, combien de jours	|__|__|__|__|__|__|
-perte du goût (agueusie)	○ Oui ○ Non
Si oui, combien de jours	|__|__|__|__|__|__|
3 - Si vous avez répondu OUI à une des questions précédentes, avez-vous eu :	
- signes respiratoires : toux, douleur de gorge, expectoration, dyspnée	○ Oui ○ Non
- signes digestifs : nausées, vomissements, diarrhée, douleur abdominale	○ Oui ○ Non
4 - Si vous avez répondu OUI à l'une des questions précédentes (2 ou 3),<br>avez-vous dû être arrêté pour ces symptômes ?	○ Oui ○ Non
Si oui, combien de jours	|__|__|__|__|__|__|
5 - Si vous avez répondu OUI à l'une des questions précédentes<br>avez-vous dû être hospitalisé pour ces symptômes ?	○ Oui ○ Non
Si oui, combien de jours	|__|__|__|__|__|__|
6 - Un diagnostic a-t-il été posé ?	○ Oui ○ Non
Si oui, lequel ?	....
7 - Avez-vous été en contact avec un cas documenté ?	○ Oui ○ Non
8- Quel est votre moyen de transport régulier pour venir au travail ?	☐ Véhicule personnel<br>☐ Transport en commun<br>☐ A pied<br>☐ Vélo/trottinette<br>☐ Autres
Préciser	....
Préciser	○ Personnel ○ Libre-service

Semaine10	
Date d'évaluation	|__|__|/|__|__|/|__|__|__|__|
Si vous ressentez, de manière significative (=entrainant une atteinte/un changement de votre qualité de vie), un des symptômes suivants,merci de contacter le PM référent. Merci de répondre aux 8 questions suivantes.	
1-Au cours de la semaine précédente, avez-vous été absent?	○ Oui ○ Non
Si oui, combien de jours	|__|__|__|__|__|__|
2 - Au cours de la semaine, avez-vous eu :	
- de la fièvre (>38°C) :	○ Oui ○ Non
Si oui, combien de jours	|__|__|__|__|__|__|
- des courbatures :	○ Oui ○ Non
Si oui, combien de jours	|__|__|__|__|__|__|
- des frissons :	○ Oui ○ Non
Si oui, combien de jours	|__|__|__|__|__|__|
- des sueurs importantes :	○ Oui ○ Non
Si oui, combien de jours	|__|__|__|__|__|__|
- des céphalées:	○ Oui ○ Non
Si oui, combien de jours	|__|__|__|__|__|__|
-perte de l'odorat (anosmie)	○ Oui ○ Non
Si oui, combien de jours	|__|__|__|__|__|__|
-perte du goût (agueusie)	○ Oui ○ Non
Si oui, combien de jours	|__|__|__|__|__|__|
3 - Si vous avez répondu OUI à une des questions précédentes, avez-vous eu :	
- signes respiratoires : toux, douleur de gorge, expectoration, dyspnée	○ Oui ○ Non
- signes digestifs : nausées, vomissements, diarrhée, douleur abdominale	○ Oui ○ Non
4 - Si vous avez répondu OUI à l'une des questions précédentes (2 ou 3), avez-vous dû être arrêté pour ces symptômes ?	○ Oui ○ Non
Si oui, combien de jours	|__|__|__|__|__|__|
5 - Si vous avez répondu OUI à l'une des questions précédentes avez-vous dû être hospitalisé pour ces symptômes ?	○ Oui ○ Non
Si oui, combien de jours	|__|__|__|__|__|__|
6 - Un diagnostic a-t-il été posé ?	○ Oui ○ Non
Si oui, lequel ?	....
7 - Avez-vous été en contact avec un cas documenté ?	○ Oui ○ Non
8- Quel est votre moyen de transport régulier pour venir au travail ?	☐ Véhicule personnel<br>☐ Transport en commun<br>☐ A pied<br>☐ Vélo/trottinette<br>☐ Autres
Préciser	....
Préciser	○ Personnel ○ Libre-service

Semaine11	
Date d'évaluation	|__|__|/|__|__|/|__|__|__|__|
Si vous ressentez, de manière significative (=entrainant une atteinte/un changement de votre qualité de vie), un des symptômes suivants,merci de contacter le PM référent. Merci de répondre aux 8 questions suivantes.	
1-Au cours de la semaine précédente, avez-vous été absent?	○ Oui ○ Non
Si oui, combien de jours	|__|__|__|__|__|__|
2 - Au cours de la semaine, avez-vous eu :	
- de la fièvre (>38°C) :	○ Oui ○ Non
Si oui, combien de jours	|__|__|__|__|__|__|
- des courbatures :	○ Oui ○ Non
Si oui, combien de jours	|__|__|__|__|__|__|
- des frissons :	○ Oui ○ Non
Si oui, combien de jours	|__|__|__|__|__|__|
- des sueurs importantes :	○ Oui ○ Non
Si oui, combien de jours	|__|__|__|__|__|__|
- des céphalées:	○ Oui ○ Non
Si oui, combien de jours	|__|__|__|__|__|__|
-perte de l'odorat (anosmie)	○ Oui ○ Non
Si oui, combien de jours	|__|__|__|__|__|__|
-perte du goût (agueusie)	○ Oui ○ Non
Si oui, combien de jours	|__|__|__|__|__|__|
3 - Si vous avez répondu OUI à une des questions précédentes, avez-vous eu :	
- signes respiratoires : toux, douleur de gorge, expectoration, dyspnée	○ Oui ○ Non
- signes digestifs : nausées, vomissements, diarrhée, douleur abdominale	○ Oui ○ Non
4 - Si vous avez répondu OUI à l'une des questions précédentes (2 ou 3), avez-vous dû être arrêté pour ces symptômes ?	○ Oui ○ Non
Si oui, combien de jours	|__|__|__|__|__|__|
5 - Si vous avez répondu OUI à l'une des questions précédentes avez-vous dû être hospitalisé pour ces symptômes ?	○ Oui ○ Non
Si oui, combien de jours	|__|__|__|__|__|__|
6 - Un diagnostic a-t-il été posé ?	○ Oui ○ Non
Si oui, lequel ?	....
7 - Avez-vous été en contact avec un cas documenté ?	○ Oui ○ Non
8- Quel est votre moyen de transport régulier pour venir au travail ?	☐ Véhicule personnel<br>☐ Transport en commun<br>☐ A pied<br>☐ Vélo/trottinette<br>☐ Autres
Préciser	....
Préciser	○ Personnel ○ Libre-service

**Semaine12**	
Date d'évaluation	|__|__|/|__|__|/|__|__|__|__|
Si vous ressentez, de manière significative (=entrainant une atteinte/un changement de votre qualité de vie), un des symptômes suivants,merci de contacter le PM référent. Merci de répondre aux 8 questions suivantes.	
1-Au cours de la semaine précédente, avez-vous été absent?	○ Oui ○ Non
Si oui, combien de jours	|__|__|__|__|__|__|
2 - Au cours de la semaine, avez-vous eu :	
- de la fièvre (>38°C) :	○ Oui ○ Non
Si oui, combien de jours	|__|__|__|__|__|__|
- des courbatures :	○ Oui ○ Non
Si oui, combien de jours	|__|__|__|__|__|__|
- des frissons :	○ Oui ○ Non
Si oui, combien de jours	|__|__|__|__|__|__|
- des sueurs importantes :	○ Oui ○ Non
Si oui, combien de jours	|__|__|__|__|__|__|
- des céphalées:	○ Oui ○ Non
Si oui, combien de jours	|__|__|__|__|__|__|
-perte de l'odorat (anosmie)	○ Oui ○ Non
Si oui, combien de jours	|__|__|__|__|__|__|
-perte du goût (agueusie)	○ Oui ○ Non
Si oui, combien de jours	|__|__|__|__|__|__|
3 - Si vous avez répondu OUI à une des questions précédentes, avez-vous eu :	
- signes respiratoires : toux, douleur de gorge, expectoration, dyspnée	○ Oui ○ Non
- signes digestifs : nausées, vomissements, diarrhée, douleur abdominale	○ Oui ○ Non
4 - Si vous avez répondu OUI à l'une des questions précédentes (2 ou 3), avez-vous dû être arrêté pour ces symptômes ?	○ Oui ○ Non
Si oui, combien de jours	|__|__|__|__|__|__|
5 - Si vous avez répondu OUI à l'une des questions précédentes avez-vous dû être hospitalisé pour ces symptômes ?	○ Oui ○ Non
Si oui, combien de jours	|__|__|__|__|__|__|
6 - Un diagnostic a-t-il été posé ?	○ Oui ○ Non
Si oui, lequel ?	....
7 - Avez-vous été en contact avec un cas documenté ?	○ Oui ○ Non
8- Quel est votre moyen de transport régulier pour venir au travail ?	☐ Véhicule personnel<br>☐ Transport en commun<br>☐ A pied<br>☐ Vélo/trottinette<br>☐ Autres
Préciser	....
Préciser	○ Personnel ○ Libre-service
